# Supplementary material for: Peripheral glia and neurons jointly regulate activity-induced synaptic remodeling at the Drosophila neuromuscular junction
Source: eLife. 2025 Nov 21;14:RP104126. doi: 10.7554/eLife.104126 (PMC12638043; doi:10.7554/eLife.104126)
Supplement: Figure 1—source data 1. [file elife-104126-fig1-data1.zip › fig 1 source data 1/Figure 1 source data 1 Fig 1A labeled.pptx]

## Slide 1
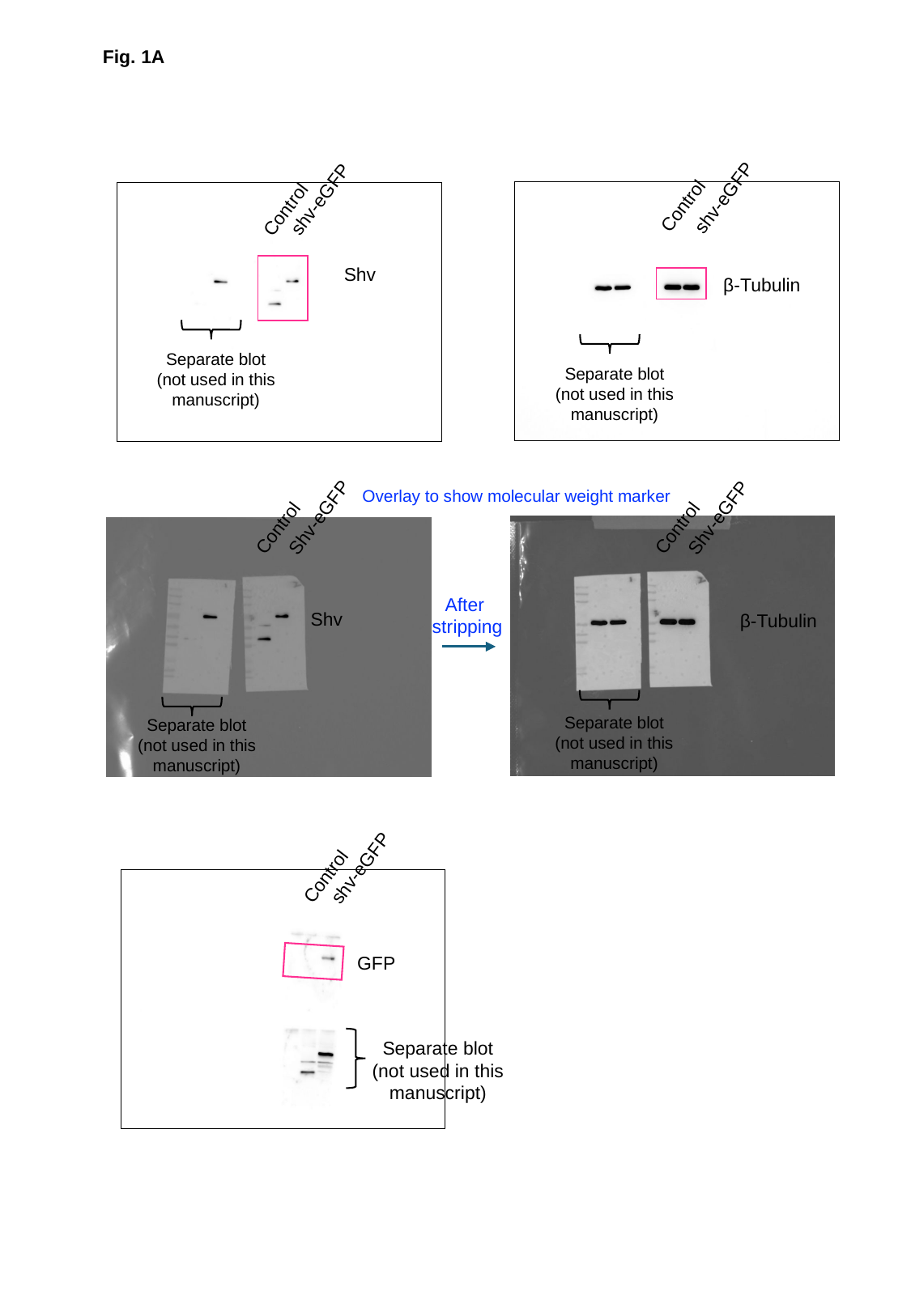

Fig. 1A
shv-eGFP
shv-eGFP
Control
Control
Shv
β-Tubulin
Separate blot
(not used in this
manuscript)
Separate blot
(not used in this
manuscript)
Overlay to show molecular weight marker
Shv-eGFP
Shv-eGFP
Control
Control
After
 stripping
Shv
β-Tubulin
Separate blot
(not used in this
manuscript)
Separate blot
(not used in this
manuscript)
shv-eGFP
Control
GFP
Separate blot
(not used in this
manuscript)
