# Supplementary figures and images for: Peripheral glia and neurons jointly regulate activity-induced synaptic remodeling at the Drosophila neuromuscular junction
Source: eLife. 2025 Nov 21;14:RP104126. doi: 10.7554/eLife.104126 (PMC12638043; doi:10.7554/eLife.104126)

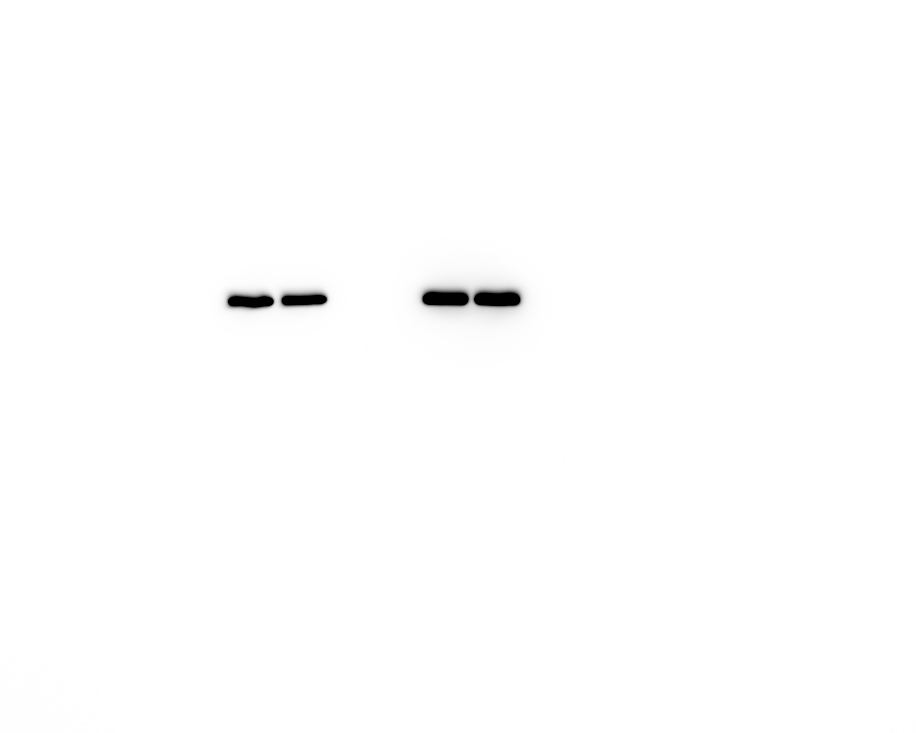

Supplement: Figure 1—source data 2. [file elife-104126-fig1-data2.zip › Figure 1- source data 2 original blots/Figure 1A source data 4 unlabeled b-tub.tif]

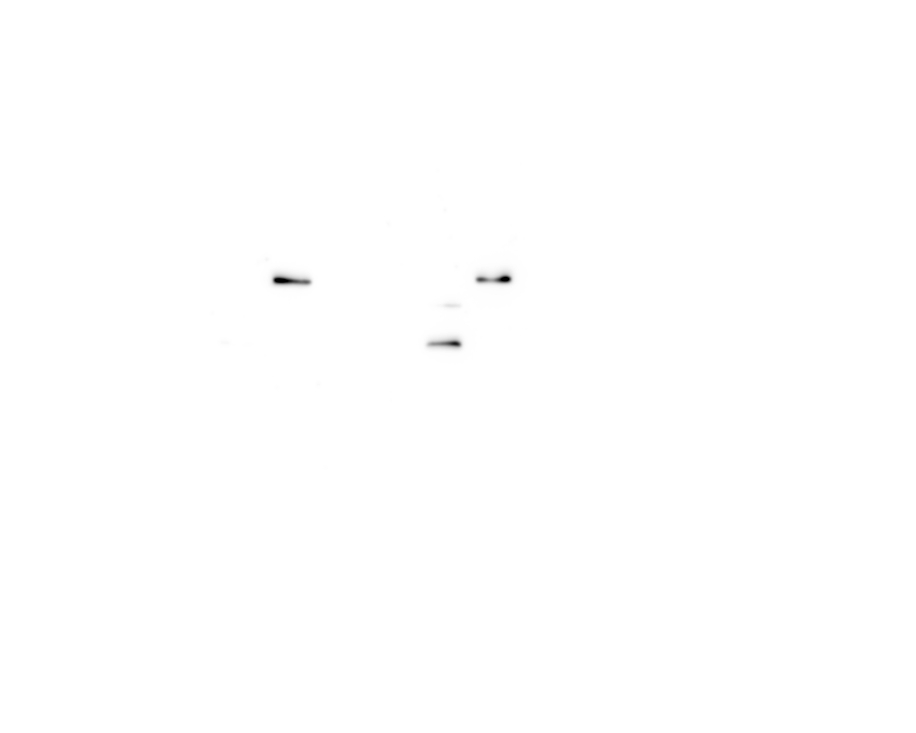

Supplement: Figure 1—source data 2. [file elife-104126-fig1-data2.zip › Figure 1- source data 2 original blots/Figure 1A - source data 3 unlabeled Shv.tif]

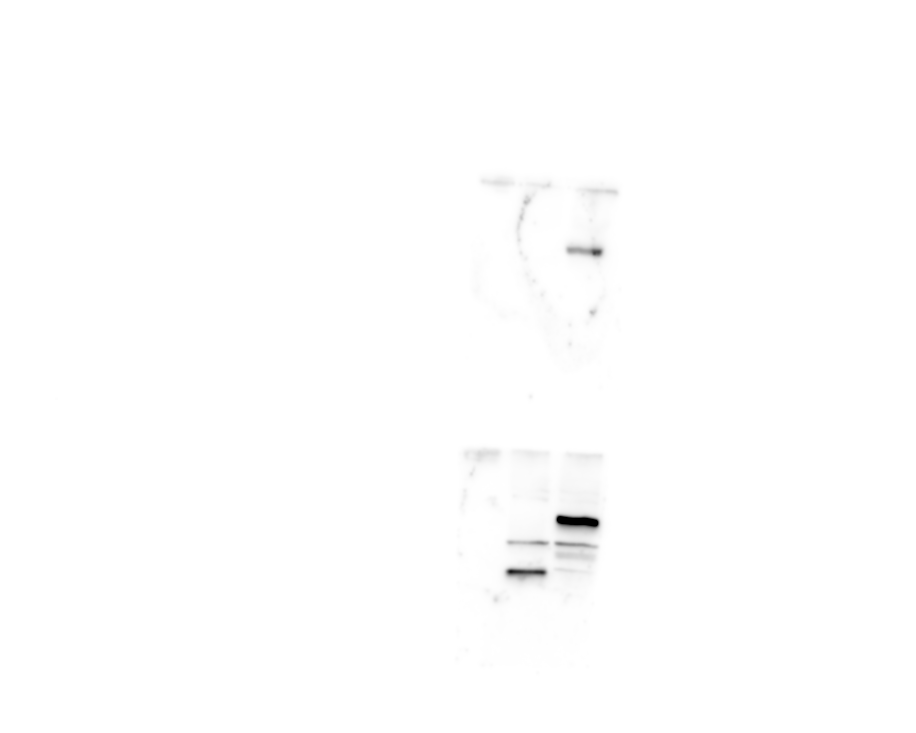

Supplement: Figure 1—source data 2. [file elife-104126-fig1-data2.zip › Figure 1- source data 2 original blots/Figure 1A - source data 2 unlabeled GFP.tif]

Fig 2- figure supplement 1B

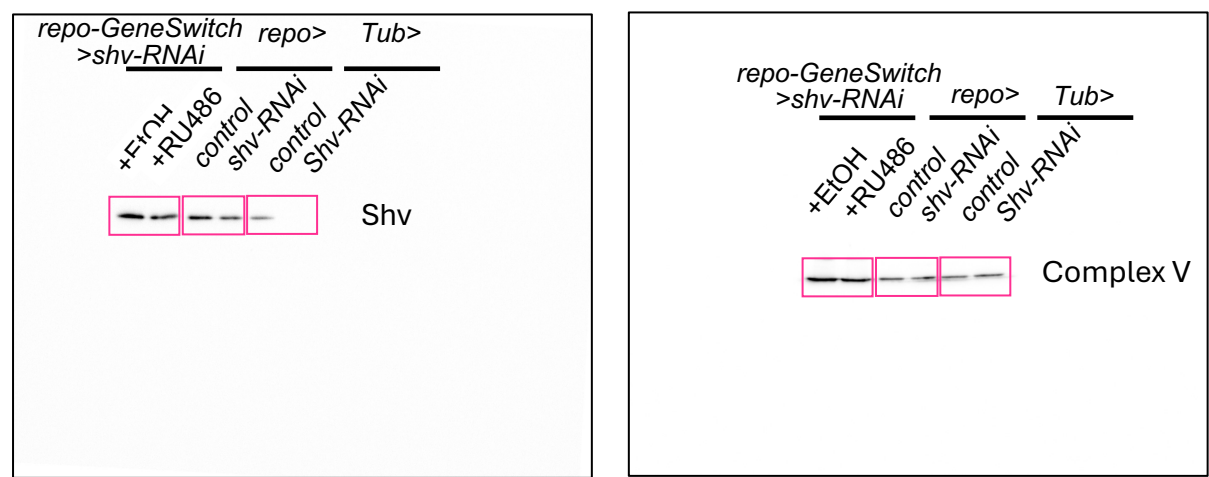

Overlay to show molecular weight marker

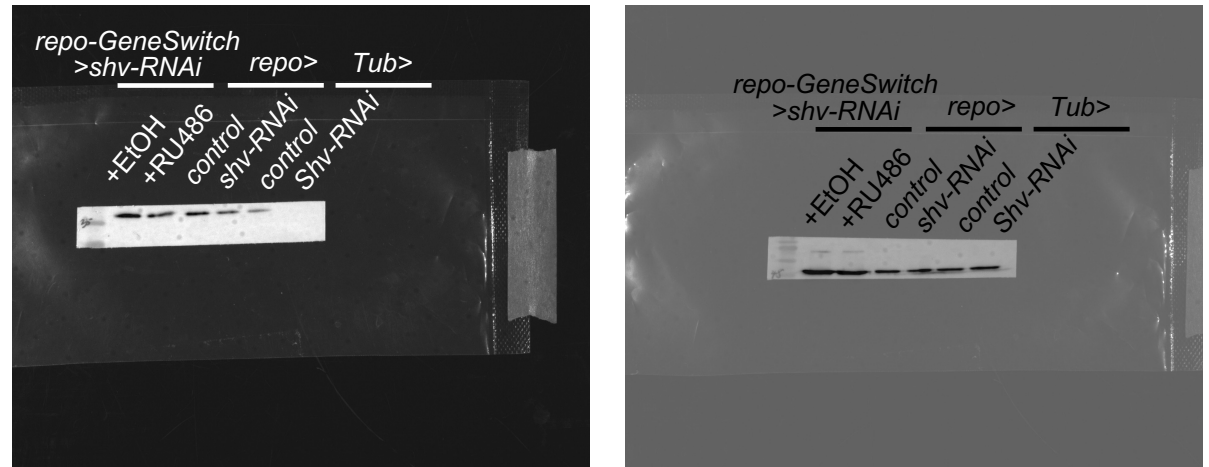

Supplement: Figure 2—figure supplement 1—source data 2. [file elife-104126-fig2-figsupp1-data2.zip › fig 2 fig suppl 1 source data 2/Figure 2 figure supplement 1 source Data 2 labeled blots.pdf]

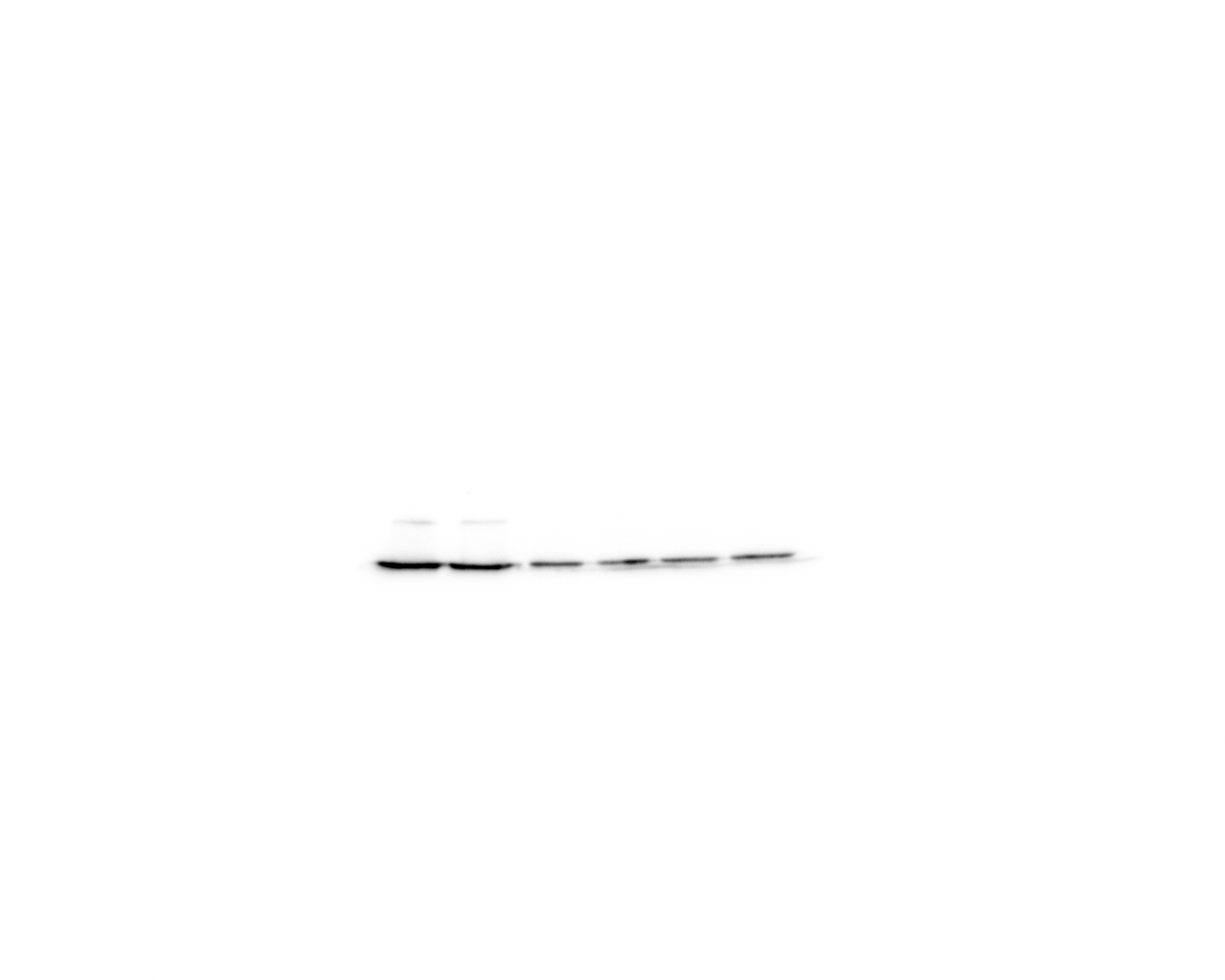

Supplement: Figure 2—figure supplement 1—source data 3. [file elife-104126-fig2-figsupp1-data3.zip › Figure 2- figure supplement -source data 3 unlabeled blots/Figure 2 - figure supplement 1-source Data 3 unlabeled complex V.tif]

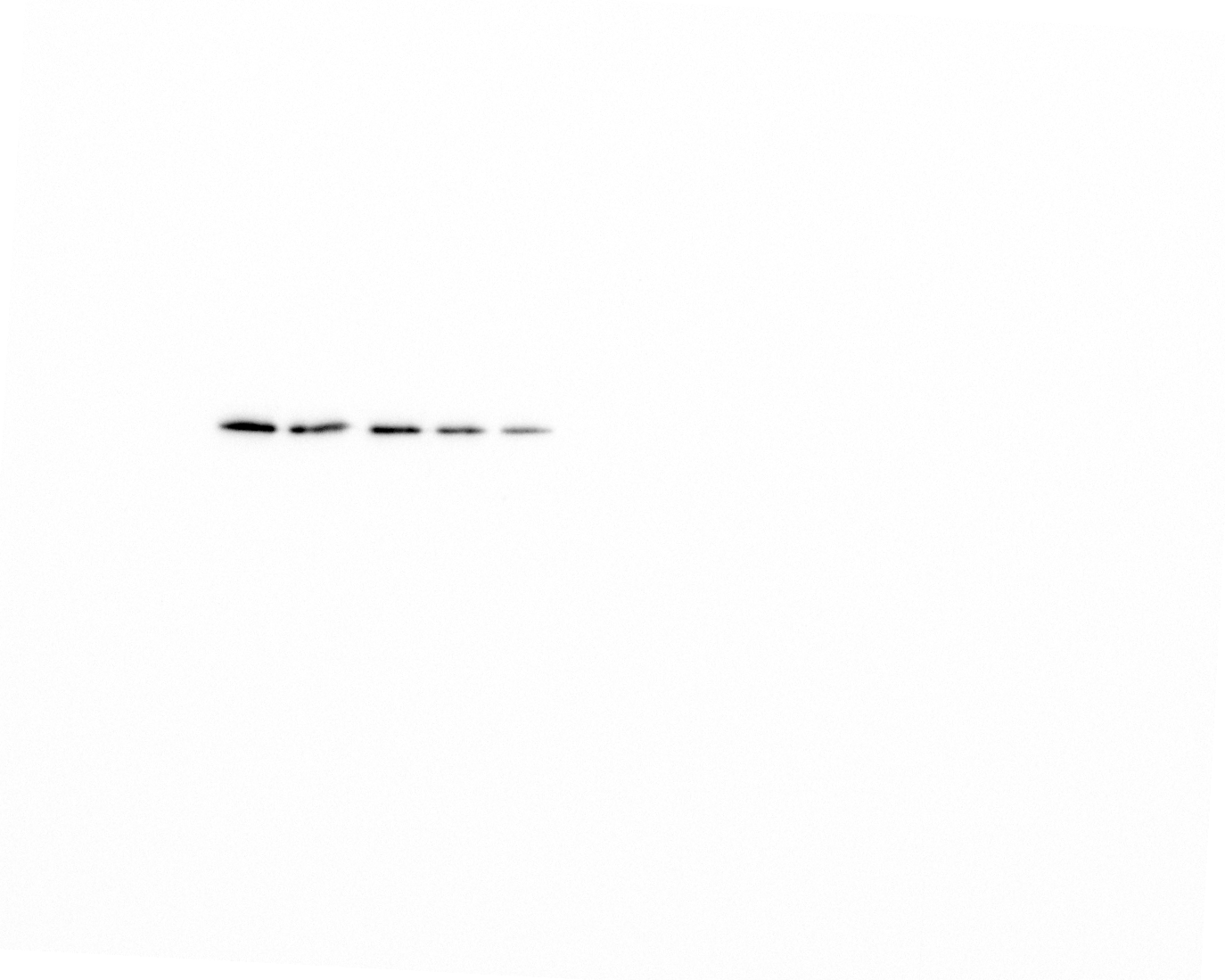

Supplement: Figure 2—figure supplement 1—source data 3. [file elife-104126-fig2-figsupp1-data3.zip › Figure 2- figure supplement -source data 3 unlabeled blots/Figure 2- figure supplement 1- source Data 2- unlabeled Shv.tif]

Fig 5 – figure supplement 1A

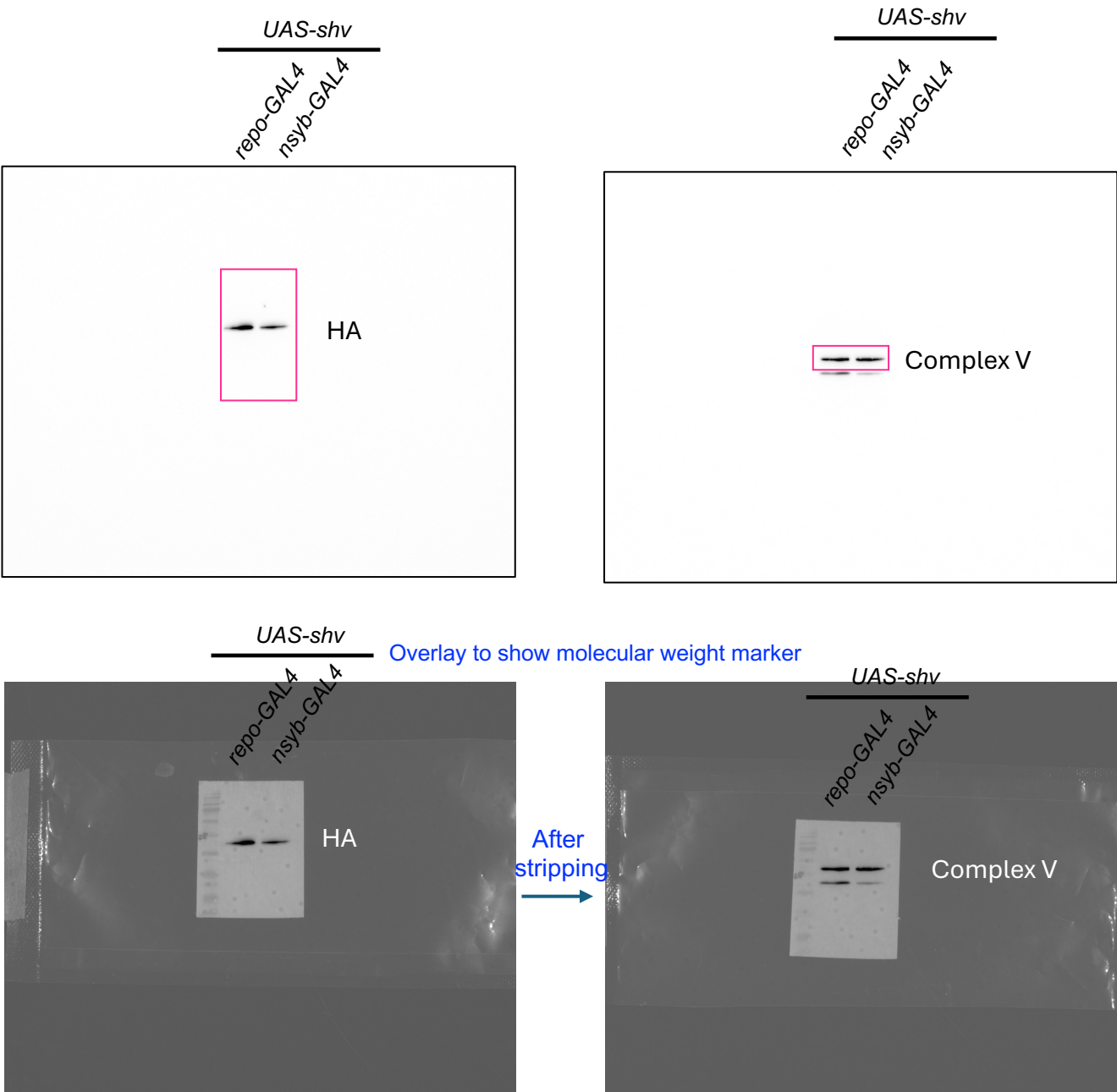

Supplement: Figure 5—figure supplement 1—source data 1. [file elife-104126-fig5-figsupp1-data1.zip › fig 5 fig suppl 1 source data 1/Figure 5 figure supplement 1 Source data 1 labeled blots.pdf]

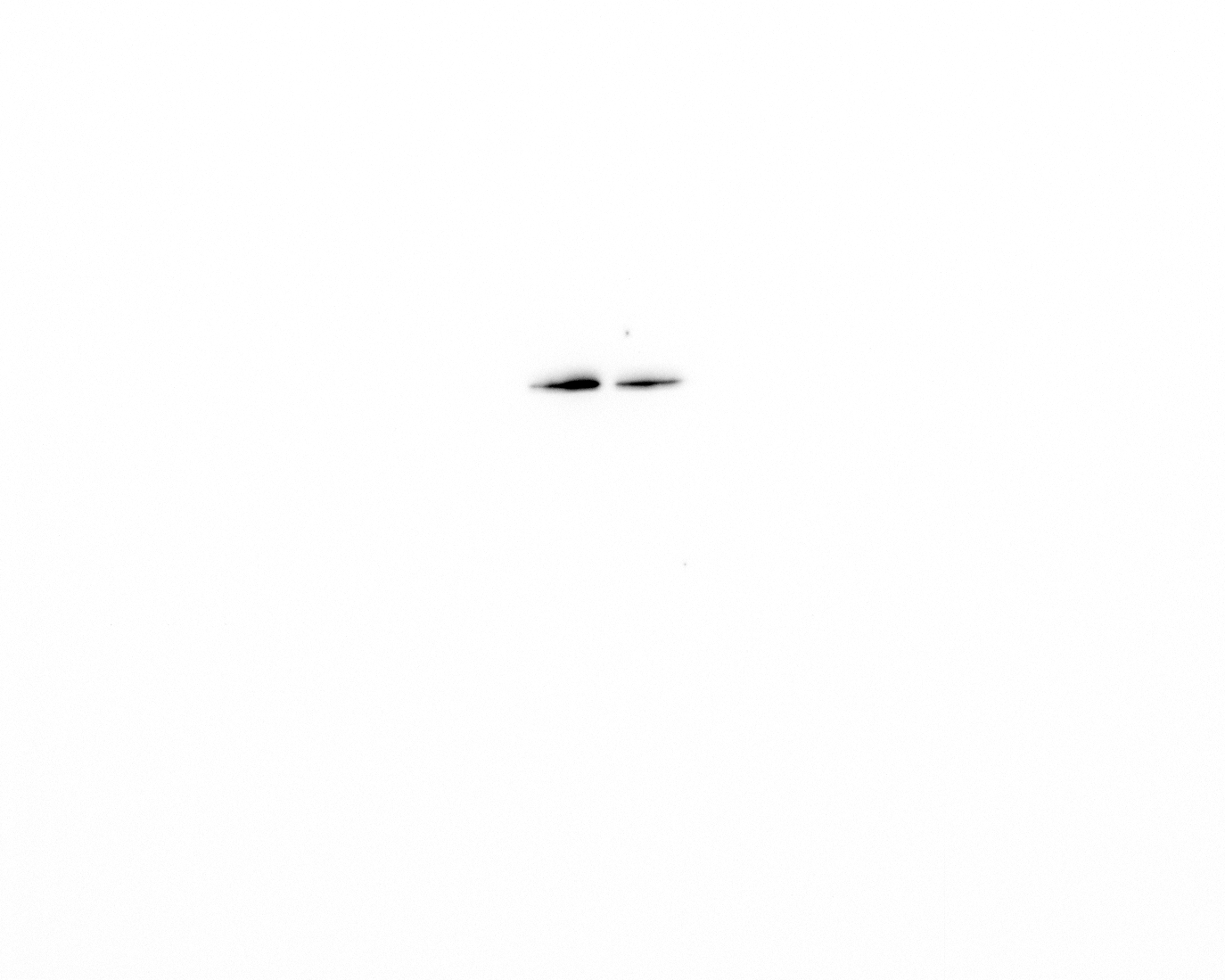

Supplement: Figure 5—figure supplement 1—source data 2. [file elife-104126-fig5-figsupp1-data2.zip › Figure 5 - figure supplement 1- Source data 2 original blots/Figure 5- figure supplement 1- source data 2 HA unlabeled.tif]

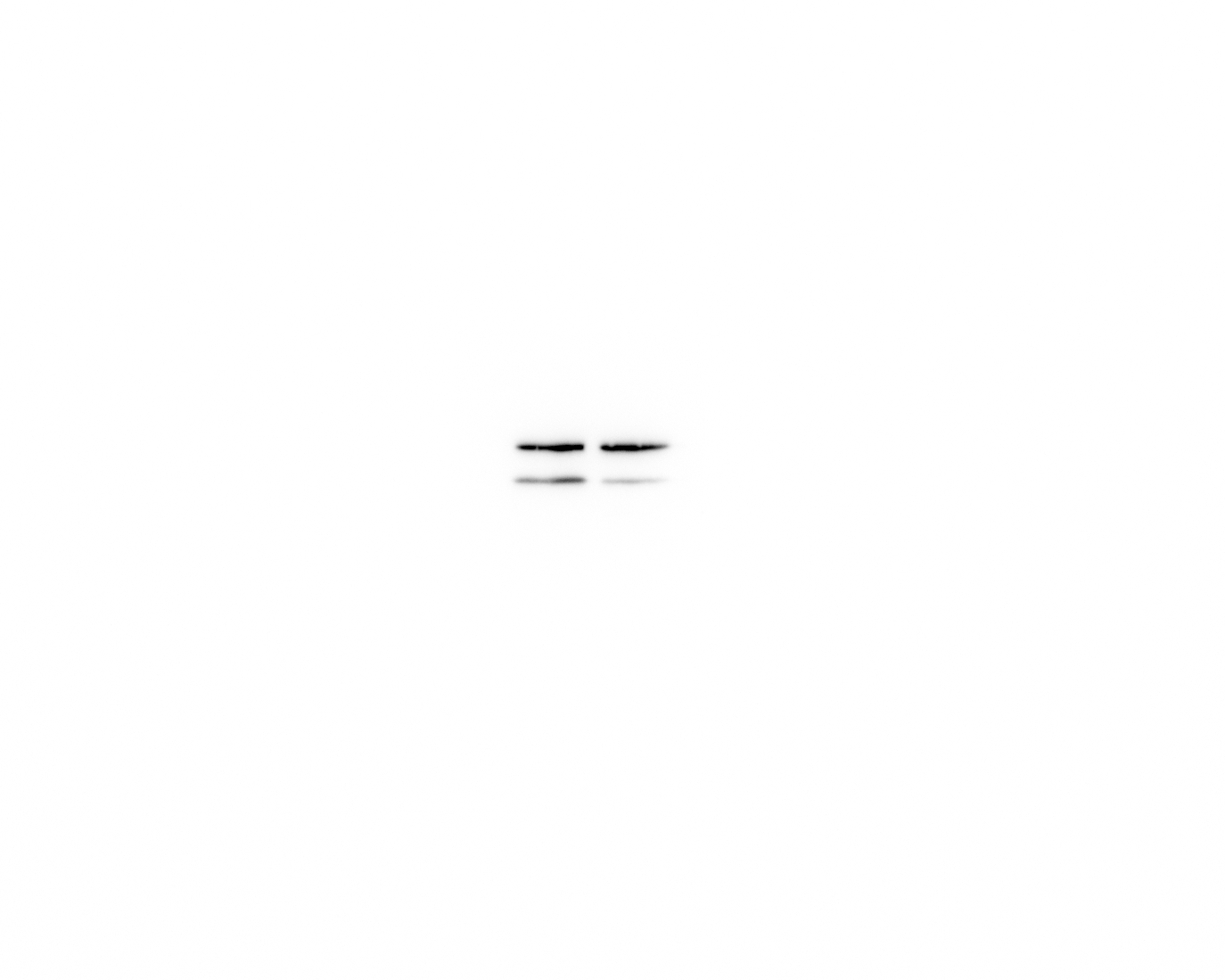

Supplement: Figure 5—figure supplement 1—source data 2. [file elife-104126-fig5-figsupp1-data2.zip › Figure 5 - figure supplement 1- Source data 2 original blots/Figure 5- figure supplement 1- source data 3 Complex V unlabeled.tif]
